# Epidemiological and Liver Biomarkers Profile of Epstein-Barr Virus Infection and Its Coinfection with Cytomegalovirus in Patients with Hematological Diseases

**DOI:** 10.3390/biom11081151

**Published:** 2021-08-04

**Authors:** Lilian Ferrari de Freitas, Jean de Melo Silva, Anderson Nogueira Barbosa, Enzo Miranda Santos, Renato Pinheiro-Silva, Gemilson Soares Pontes

**Affiliations:** 1 Programa de Pós-graduação em Hematologia, Universidade do Estado do Amazonas, Manaus 69050-010, Brazil; lilianferrarif@gmail.com (L.F.d.F.); enzomiranda21@gmail.com (E.M.S.); fernandesmp3@gmail.com (R.P.-S.); 2 Programa de Pós-graduação em Imunologia Básica e Aplicada, Universidade Federal do Amazonas (UFAM), Manaus 69067-005, Brazil; biomedicojean@gmail.com; 3 Coordenação Sociedade, Ambiente e Saúde, Laboratório de Virologia e Imunologia, Instituto Nacional de Pesquisa da Amazônia (INPA), Manaus 69067-375, Brazil; anderson.nb6@gmail.com

**Keywords:** Epstein-Barr, cytomegalovirus, coinfection, prevalence, ferritin, hematological diseases

## Abstract

Epstein-Barr virus (EBV) and cytomegalovirus (CMV) are viruses globally distributed that have been associated with the development and prognosis of many pathologies, including hematological diseases. This study aimed to characterize the epidemiological profile of EBV infection and the infection-correlated hepatic manifestations in patients with hematological diseases of *the northern Brazilian state of Amazonas*. A total of 228 patients were serologically tested for the presence of anti-EBV and anti-CMV IgG antibodies through an enzyme-linked immunosorbent assay. The coinfection with CMV, sociodemographic and laboratory records of all patients were also assessed. The overall prevalence observed among the study population for EBV infection and EBV/CMV coinfection was 85.09% (95% CI: 0.80–0.90) and 78.51% (95% CI: 0.73–0.84), respectively. The age group 31–40 years old were more susceptible to EBV/CMV coinfection (95% CI: 1.59–93.41, *p* = 0.011), while young people aged 1–10 years old were less affected for both EBV infection (CI 95%; 0.66–0.91, *p* = 0.001) and EBV/CMV coinfection (95% CI: 0.52–0.81, *p* < 0.0001). High serum levels of the liver biomarker ferritin were associated with EBV infection (95% CI: 1.03–1.54, *p* = 0.031) and EBV/CMV coinfection (95% CI: 1.02–1.70, *p* = 0.038). Our findings indicated that the elevated prevalence of EBV infection is not associated with the hematological diseases or transfusion rates, but with the socioeconomic status of the study population. Also, this study suggests that the EBV infection and its coinfection with CMV are related to the increase of serum ferritin levels.

## 1. Introduction

Epstein-Barr virus (EBV) and cytomegalovirus (CMV) belong to the *Herpesviridae* family and affect over 90% of the global population. Both viruses are linked to different malignancies, such as hematological diseases [1]. Coinfection between EBV and CMV is frequent and the prevalence rates of EBV infection fluctuate according to population, geographical region and socioeconomic status [2].

High rates of EBV infection have been reported in individuals with lower socioeconomic status, especially among non-white-skinned individuals and low-income families [3,4]. In children, the EBV infection prevalence may range from 20 to 80%, with the highest rates occurring in developing countries [4,5]. Even though most EBV or CMV-infected individuals remain asymptomatic throughout life, these viruses are the main cause of infectious mononucleosis in immunocompromised patients [6].

EBV infection has been associated with liver damage in several stages [7]. The liver is one of the first organs involved in the EBV infection cycle. High levels of serum glutamic pyruvic transaminase (SGPT) and serum glutamic oxaloacetic transaminase (SGOT) in infected individuals may indicate EBV-induced liver injury [8,9]. A study case reported high serum levels of ferritin and lactate dehydrogenase (LDH) in an 18 years old girl carrier of EBV-related infectious mononucleosis [10]. The girl was also a carrier of autoimmune hemolytic anemia, which could be caused by the EBV infection.

The association of EBV with hematological diseases was first described in patients with Burkitt’s lymphoma [11]. Afterward, the EBV infection was also linked to the pathogenesis of other hematological diseases, such as Hodgkin lymphoma, leukemia and post-transplant lymphomas [12,13,14]. However, the impact of EBV infection on patients with hematological diseases is not completely understood.

In this study, we evaluated the epidemiological and liver biomarkers profile of EBV infection and its coinfection with CMV in individuals from the *northern Brazilian state of Amazonas who have hematological diseases.*

## 2. Materials and Methods

### 2.1. Study Population

This project was approved by the Human Research Ethics Committee of the Hematology and Hemotherapy Hospital Foundation of Amazonas (approval number: 4.191.868), following the resolution number 466/2012 of the Brazilian National Health Council. From September 2020 to March 2021, a total of 228 patients suffering from anemia, lymphoma, leukemia or platelet diseases were serologically tested for the presence of anti-EBV and anti-CMV IgG antibodies (Abs). An epidemiological questionnaire was administered to collect patient’s social demographics information. The study population was composed of individuals of either gender and from different ethnicities who were aged from 1 to 92 years old. Clinical data records of patients were assessed through the database of Brazil’s diagnoses (www.diagnosticosdobrasil.com.br, accessed on 20 April 2021).

### 2.2. Serological Analysis

Serum samples of patients were tested for the presence of anti-EBV or anti-CMV IgG Abs, using an immunoenzymatic assay (EBV/CMV-VCA IgG, Euroimmun, Lubeck, Germany), which was performed following the manufacturer’s recommendations. The optical density resulting from the test was measured in a spectrophotometer (Molecular Devices, San Jose, CA, USA) at a 450 nm filter. The positivity of the test was estimated according to the cut-off formula indicated by the manufacturer (antibody ratio of control or patient sample/ratio of calibrator).

### 2.3. Statistical Analysis

Descriptive statistical analysis was used to evaluate the sociodemographic variables and serum levels of anti-EBV or anti-CMV IgG Abs. The association between sociodemographic characteristics (gender, age, ethnicity, marital status, income, level of schooling) and prevalence of EBV infection or EBV/CMV coinfection were evaluated through the prevalence ratio (PR) analysis, using Koopman asymptotic score and chi-square with Yates’ correction. The influence of transfusion rates on the prevalence of EBV infection or EBV/CMV coinfection was also assessed through PR analysis. Pearson’s correlation (r) was used to measure the degree linear relationship among liver biomarkers in the serum of EBV-infected patients. We considered strong positive and negative correlations when r > 0.7 or r < −0.7, respectively. All statistical analyses were performed using GraphPad Prism v8.0.1 software. In all analyses, a value of *p* < 0.05 was considered statistically significant.

## 3. Results

### 3.1. Prevalence of EBV Infection and EBV/CMV Coinfection

A total of 194 patients (85.09%; 95% CI: 0.80–0.90) showed positivity for anti-EBV IgG antibody and 179 (78.51%; 95% CI: 0.73–0.84) were positive for EBV/CMV coinfection. Patients with immune thrombocytopenic purpura (ITP) had the highest prevalence rates for EBV infection (90%) and EBV/CMV coinfection (80%). No direct relationship was observed between the susceptibility for EBV infection or EBV/CMV coinfection and the type of hematological disease.

The age group > 60 years old showed the highest prevalence (100%) while the age group 31–40 years old were more susceptible to coinfection (95% CI: 1.59–93.41, *p* = 0.011). The group 1–10 years old were less affected for both EBV infection (95% CI: 0.66–0.91, *p* = 0.001) and EBV/CMV coinfection (95% CI: 0.52–0.81, *p* < 0.0001), showing prevalence rates of 71.88% and 57.81%, respectively (Table 1).

Most of the patients (64.04%) had a low level of education. Over 39% of the study population was illiterate or literate. Illiterate patients were less susceptible to EBV infection (95% CI: 0.60–0.94, *p* = 0.004) and EBV/CMV coinfection (95% CI: 0.55–0.93, *p* = 0.005). Literate patients were only less susceptible to EBV/CMV coinfection (95% CI: 0.66–0.99, *p* = 0.026). The majority of individuals in these groups (illiterate and literate) were within the age range between 1–10 years and, due to the severity of hematological diseases, these patients were not attending school regularly (Table 1). The correlation between the rate of transfusion of blood products and the prevalence of EBV infection or EBV/CMV coinfection was not statically significant (Table 2).

### 3.2. Liver Biomarkers Profile

We assessed the levels of serum glutamic pyruvic transaminase (SGPT), serum glutamic oxaloacetic transaminase (SGOT), *lactate dehydrogenase* (LDH) and ferritin of the study population (Table 3). The results demonstrated an association between elevated serum levels of ferritin and EBV infection or EBV/CMV coinfection. From the total of 28 patients who showed increased (altered) serum levels of ferritin, 27 (96.43%) tested positive for EBV (95% CI: 1.15–89.16, *p* = 0.031) and 25 patients (89.29%) were coinfected with CMV (95% CI: 1.02–1.70, *p* = 0.038).

**Figure 1:**
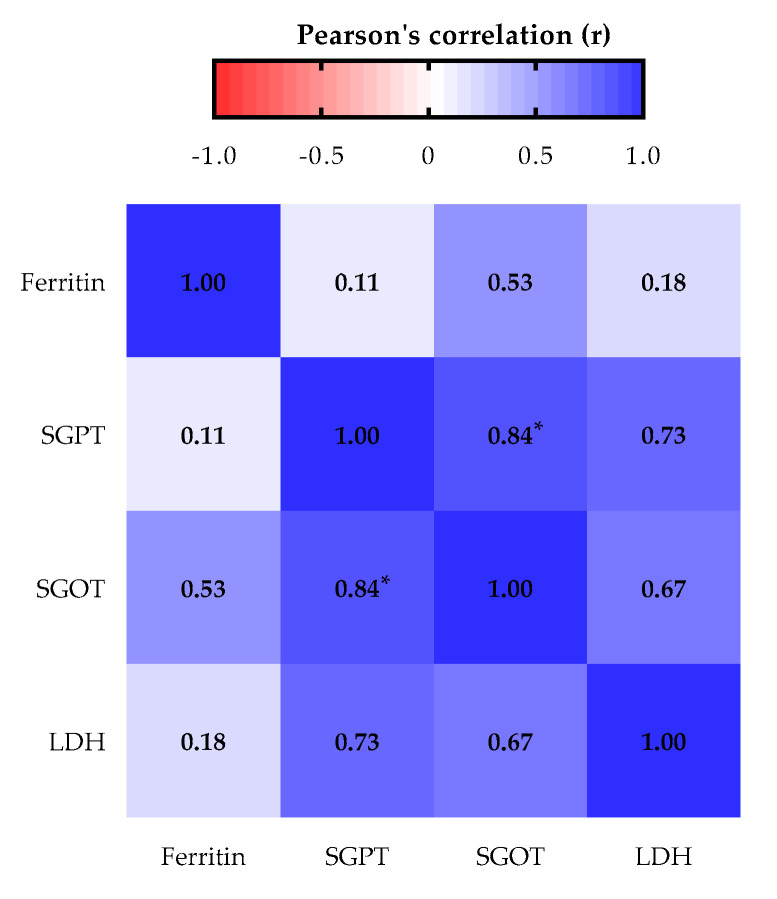
Pearson’s correlation between the levels of liver biomarkers in patients with altered ferritin. Values of r > 0.7 and r < −0.7 were considered strong positive and negative correlations, respectively. * *p* < 0.05.

When we analyzed the correlation between the levels of SGPT, SGOT, LDH and ferritin in individuals with increased serum levels of ferritin, only positive correlations were verified. The serum levels of SGPT and LDH were strongly correlated. As expected, SGPT and SGOT showed a significant positive correlation (r = 0.84, *p* = 0.039). Both correlations demonstrated that the increase or decrease in the serum levels of these biomarkers happens in the same direction (Figure 1).

## 4. Discussion

The herpesviruses CMV and EBV are pathogens globally disseminated [15,16]. Nevertheless, only a few studies investigate the EBV/CMV coinfection and its impact on patients with hematological diseases. In this study, the EBV infection and its coinfection with CMV were found to be highly prevalent among individuals of the Brazilian northern state of Amazonas who suffer from hematological diseases. The prevalence of EBV/CMV coinfection (78.51%) observed in this study was greater than the one found in the healthy adult population (66.02%) from the same region of the study population [17]. The present study suggests that patients with hematological diseases are possibly more susceptible to EBV/CMV coinfection.

Our findings demonstrated that the prevalence of EBV infection increased with age. Adults aged 60 years and older showed the highest prevalence rates, while children aged 1–10 years old showed the lowest. Patients aged 31–40 years old were more susceptible to EBV/CMV coinfection. Similar results were found among patients carriers of infectious mononucleosis in the United Kingdom and healthy individuals from the Brazilian state of Espirito Santo [5,18]. However, it is important to point out that the children of our study population were often absent from school owing to their health condition, which could directly influence the prevalent rates, considering that daycare and school attendance are important factors implicated in the mechanism of EBV and CMV transmission among children [4,19].

The study population had a low level of education. Previous studies reported a high prevalence of EBV infection among families with education-related deficiencies [5,20]. However, in our study, illiterate patients were less susceptible to EBV infection and EBV/CMV coinfection. This might have occurred because most of the illiterate patients were children who were not attending school or daycare centers regularly.

We found no correlation between EBV infection and gender. A study conducted in Iran observed an increased prevalence rate of EBV infection among men [21]. Although both men and women showed similar prevalences in our study, we observed that married individuals were more susceptible to EBV infection and EBV/CMV coinfection. Nonetheless, we did not evaluate the sexual behavior of the study population to establish the causal link between marital status and the prevalence rates.

EBV and CMV infections have been associated with several hematological diseases, especially regarding the progression of hemoglobinopathies, lymphomas, myelomas, hemophilia, sickle cell and aplastic anemia diseases [22]. In this study, patients carriers of ITP showed the highest prevalence rates for EBV infection and EBV/CMV coinfection. It is known that both EBV and CMV can trigger the development of ITP disease [23]. A retrospective study that investigated EBV-associated ITP in 108 children observed the presence of acute EBV infection in 32.4% of the patients [24]. In this study, we did not evaluate the influence of EBV in the development or prognosis of ITP disease or other hematological diseases. However, it is imperative to perform longitudinal studies to assess the impact of EBV infection or EBV/CMV coinfection on hematological diseases, especially because these infections have been implicated in many physiological complications that might affect the development or the clinical course of these diseases [2,23].

Kogawa et al. (2014) reported high serum levels of bilirubin and ferritin in EBV-infected children carriers of hemophagocytic lymphohistiocytosis, which worsened the prognosis of the disease [25]. Also, increased levels of serum ferritin were found in CMV-infected individuals and patients with acute mononucleosis [26,27,28,29]. In this study, high levels of serum ferritin were associated with EBV infection and EBV/CMV coinfection. Over 90% of patients with altered levels of serum ferritin were positive for EBV infection or EBV/CMV coinfection. Thus, our findings suggest that altered levels of serum ferritin could work as a liver biomarker that indicates the presence of EBV infection or EBV/CMV coinfection, especially in the context of hematological diseases. However, additional studies are needed to better characterize this possible correlation.

We observed no association between blood transfusion rates and susceptibility to EBV infection. Indeed, blood transfusions have been indicated as an important risk factor for the transmission of EBV, especially among immunosuppressed individuals [30]. In this study, we had no access to the patients’ clinical records regarding their immunological status. Other limitations of this study were the restricted number of patients, the unsatisfactory patient clinical records available, and the project budget constraints that did not allow to perform the viral genotyping.

In conclusion, our findings demonstrated that EBV infection is widely disseminated among individuals of the northern Brazilian state of Amazonas who suffer from hematological diseases. Increasing levels of serum ferritin are either associated with EBV infection or EBV/CMV coinfection. Married patients aged 31–40 years old were more susceptible to EBV infection and EBV/CMV coinfection, while children aged 1–10 years were less affected. Our data provide a new contribution to the epidemiology of EBV infection and EBV/CMV coinfection and their influence over liver function in the context of hematological diseases.

## Figures and Tables

**Table 1:** Prevalence of EBV infection and EBV/CMV coinfection according to sociodemographic characteristics of the study population.

Sociodemographic Characteristics	N (%)	EBV IgG Positive (%)	EBV PR (95% CI)	*p*-Value ^a^	Coinfection EBV/CMV Positive (%)	Coinfection PR (95% CI)	*p*-Value ^a^
**Gender**							
Male	112 (49.12)	92 (82.14)	0.93 (0.83–1.04)	0.149	84 (75.00)	0.92 (0.79–1.05)	0.134
Female	116 (50.88)	102 (87.93)	1.00 (ref.)		95 (81.90)	1.00 (ref.)	
**Age**							
1–10	64 (28.07)	46 (71.88)	0.80 (0.66–0.91)	0.001 *	37 (57.81)	0.67 (0.52–0.81)	<0.0001 *
11–20	52 (22.81)	45 (86.54)	1.00 (ref.)		41 (78.85)	1.00 (ref.)	
21–30	28 (12.28)	26 (92.86)	1.11 (0.92–1.22)	0.171	25 (89.29)	1.16 (0.94–1.31)	0.108
31–40	29 (12.72)	28 (96.55)	1.16 (0.99–1.26)	0.058	28 (96.55)	1.27 (1.08–1.40)	0.011 *
41–50	20 (8.77)	15 (75.00)	0.87 (0.62–1.05)	0.159	15 (75.00)	0.95 (0.67–1.15)	0.454
51–60	20 (8.77)	19 (95.00)	1.13 (0.90–1.23)	0.165	18 (90.00)	1.16 (0.90–1.31)	0.153
>60	15 (6.58)	15 (100.00)			15 (100.00)		
**Ethnicity**							
White	64 (28.07)	54 (84.38)	0.99 (0.85–1.10)	0.493	49 (76.56)	0.97 (0.81–1.11)	0.395
Brown	146 (64.04)	125 (85.62)	1.00 (ref.)		115 (78.77)	1.00 (ref.)	
Black	18 (7.89)	15 (83.33)	0.98 (0.71–1.13)	0.450	15 (83.33)	1.07 (0.77–1.24)	0.413
**Marital status**							
Single	168 (73.68)	137 (81.55)	1.00 (ref.)		124 (73.81)	1.00 (ref.)	
Married	60 (26.32)	57 (95.00)	1.17 (1.04–1.28)	0.011 *	55 (91.67)	1.24 (1.09–1.40)	0.003 *
**Income**							
Up to a minimum wage	115 (50.44)	98 (85.22)	1.02 (0.90–1.12)	0.448	90 (78.26)	0.99 (0.86–1.14)	0.472
2–5 minimum wages	102 (44.74)	85 (83.33)	1.00 (ref.)		78 (76.47)	1.00 (ref.)	
Above 6 minimum wages	11 (4.82)	11 (100.00)			11 (100.00)		
**Level of schooling**							
Illiterate **	36 (15.79)	25 (69.44)	0.79 (0.60–0.94)	0.004 *	22 (61.11)	0.75 (0.55–0.93)	0.005 *
Literate ***	53 (23.25)	42 (79.25)	0.91 (0.76–1.04)	0.127	36 (67.92)	0.83 (0.66–0.99)	0.026 *
Complete middle school	57 (25.00)	52 (91.23)	1.10 (0.96–1.21)	0.099	49 (85.96)	1.13 (0.97–1.28)	0.081
Complete high school	49 (21.49)	45 (91.84)	1.10 (0.96–1.22)	0.102	43 (87.76)	1.16 (0.98–1.31)	0.057
Undergraduate	23 (10.09)	21 (91.30)	1.00 (ref.)		21 (91.30)	1.00 (ref.)	

PR: Prevalence ratio; CI = Confidence interval; ^a^ Chi-square with Yates’ correction; * *p* < 0.05; Prevalence Risk CI calculated by the method Koopman asymptotic score; ** people unable to read and write; *** people able to read and write; All individuals in both the groups literate and illiterate were not attending school; Brazilian national minimum monthly wage: approximately USD 192 dollars.

**Table 2:** Prevalence of EBV infection and EBV/CMV coinfection according to the type of hematological diseases and the rates of transfusion in a year.

Hematological Disease/Transfusion	N (%)	EBV IgG Positive (%)	EBV PR (95% CI)	*p*-Value ^a^	Coinfection Positives (%)	Coinfection PR (95% CI)	*p*-Value ^a^
**Hematological disease**							
Anemia	72 (31.58)	61 (84.72)	0.99 (0.87–1.11)	0.462	56 (77.78)	0.99 (0.84–1.13)	0.496
ITP	20 (8.77)	18 (90.00)	1.06 (0.82–1.19)	0.376	16 (80.00)	1.02 (0.74–1.21)	0.454
Leukemia	117 (51.32)	98 (83.76)	0.97 (0.86–1.08)	0.348	92 (78.63)	1.00 (0.87–1.16)	0.454
ALL	83 (70.94)	68 (81.93)	0.93 (0.80–1.14)	0.286	65 (78.31)	0.99 (0.82–1.26)	0.454
AML	22 (18.80)	19 (86.36)	1.00 (ref.)		17 (77.27)	1.00 (ref.)	
CLL	1 (0.86)	1 (100.00)			1 (100.00)		
CML	11 (9.40)	10 (90.91)	1.10 (0.75–1.26)	0.403	9 (81.82)	1.05 (0.66–1.28)	0.454
Lymphoma	19 (8.33)	17 (89.47)	1.00 (ref.)		15 (78.95)	1.00 (ref.)	
**Type of transfusion**	143 (62.72)	119 (83.22)	0.94 (0.85–1.06)	0.202	111 (77.62)	0.97 (0.85–1.13)	0.399
Complete	101 (70.63)	82 (57.34)	0.92 (0.80–1.10)	0.223	76 (75.25)	0.90 (0.76–1.11)	0.202
Erythrocytes	30 (20.98)	28 (19.58)	1.16 (0.96–1.31)	0.082	26 (86.67)	1.15 (0.92–1.35)	0.138
Erythrocytes/Platelets	2 (1.40)	2 (1.40)			2 (100.00)		
Plasma	1 (0.70)	0 (0.00)					
Platelets	9 (6.29)	7 (4.90)	0.93 (0.54–1.15)	0.496	7 (77.78)	1.00 (0.58–1.25)	0.344
No transfusion	85 (37.28)	75 (88.24)	1.00 (ref.)		68 (80.00)	1.00 (ref.)	0.399

PR = Prevalence ratio; CI = Confidence interval; ^a^ Chi-square with Yates’ correction; Prevalence Risk CI calculated by the method Koopman asymptotic score; ALL = Acute Lymphocytic Leukemia; AML = Acute Myeloid leukemia; CLL = Chronic Lymphocytic Leukemia; CML = Chronic Myeloid Leukemia.

**Table 3:** Serum level of liver biomarkers according to positivity for EBV infection or EBV/CMV coinfection.

Liver Biomarkers	N (%)	EBV IgG Positive (%)	EBV PR (95% CI)	*p*-Value ^a^	Coinfection EBV/CMV Positive (%)	Coinfection PR (95% CI)	*p*-Value ^a^
**Ferritin**							
Altered	28 (38.89)	27 (96.43)	1.25 (1.03–1.54)	0.031 *	25 (89.29)	1.31 (1.02–1.70)	0.038 *
Normal	44 (61.11)	34 (77.27)	1.00 (ref.)		30 (68.18)	1.00 (ref.)	
**SGPT**							
Altered	7 (20.59)	6 (85.71)	1.16 (0.64–1.63)	0.442	5 (71.43)	1.02 (0.49–1.56)	0.341
Normal	27 (79.41)	20 (74.07)	1.00 (ref.)		19 (70.37)	1.00 (ref.)	
**SGOT**							
Altered	13 (34.21)	10 (76.92)	1.07 (0.66–1.56)	0.476	10 (76.92)	1.20 (0.73–1.84)	0.328
Normal	25 (65.79)	18 (72.00)	1.00 (ref.)		16 (64.00)	1.00 (ref.)	
**LDH**							
Altered	20 (55.56)	18 (90.00)	12.00 (0.88–1.81)	0.227	17 (85.00)	1.24 (0.86–1.96)	0.223
Normal	16 (44.44)	12 (75.00)	1.00 (ref.)		11 (68.75)	1.00 (ref.)	
**Creatinine**							
Altered	24 (64.86)	18 (75.00)			17 (70.83)		
Normal	13 (35.14)	13 (100.00)			13 (100.00)		
**Liver function**							
Altered	40 (69.00)	34 (85.00)	1.16 (0.93–1.41)	0.126	32 (80.00)	1.18 (0.93–1.49)	0.123
Normal	68 (31.00)	50 (73.53)	1.00 (ref.)		46 (67.65)	1.00 (ref.)	

PR = Prevalence ratio; CI = Confidence interval; ^a^ Chi-square with Yates’ correction; * *p* < 0.05; Prevalence Ratio CI calculated by the Koopman asymptotic score method; Normal reference values: Ferritin (man = 23.9–336.2, woman = 11–306.8 ng/mL); B12 vitamin (130–868 pg/mL); SGPT (man < 41 U/L, woman < 33 U/L); SGOT (man < 40 U/L, woman < 32 U/L); LDH (< 246 U/L); Creatinine (0.51–0.95 mg/dL).
